# Identification of a Plasma Exosomal lncRNA‐ and circRNA‐Based ceRNA Regulatory Network in Patients With Lung Adenocarcinoma

**DOI:** 10.1111/crj.70026

**Published:** 2024-10-20

**Authors:** Wangyu Zhu, Huafeng Zhang, Liwei Tang, Kexin Fang, Nawa Lin, Yanyan Huang, Yongkui Zhang, Hanbo Le

**Affiliations:** ^1^ Cell and Molecular Biology Laboratory Zhoushan Hospital of Wenzhou Medical University Zhoushan Zhejiang China; ^2^ Lung Cancer Research Centre Zhoushan Hospital of Wenzhou Medical Zhoushan Zhejiang China; ^3^ Department of Cardio‐Thoracic Surgery Zhoushan Hospital of Wenzhou Medical Zhoushan Zhejiang China

**Keywords:** ceRNA, circRNA, exosome, lncRNA, lung adenocarcinoma, plasma

## Abstract

**Background:**

Exosomes have been established to be enriched with various long noncoding RNAs (lncRNAs) and circular RNAs (circRNAs) that exert various biological effects. However, the lncRNA‐ and circRNA‐mediated coexpression competing endogenous RNA (ceRNA) regulatory network in exosomes derived from the plasma of patients with lung adenocarcinoma (LUAD) remains elusive.

**Methods and Results:**

This study enrolled nine patients with lung adenocarcinoma and three healthy individuals, and the differential expression of messenger RNAs (mRNAs), lncRNAs, and circRNAs was detected using microarray analysis, while microRNAs (miRNAs) were detected through RNA sequencing. Additionally, bioinformatics algorithms were applied to evaluate the lncRNA–miRNA–mRNAs/circRNA–miRNA–mRNA network. Differentially expressed cicRNAs were identified via quantitative reverse transcription polymerase chain reaction (RT‐qPCR). A total of 1016 lncRNAs, 1396 circRNAs, 45 miRNAs, and 699 mRNAs were differentially expressed in the plasma exosomes of patients with LUAD compared with healthy controls. Among them, 881 lncRNAs were upregulated and 135 were downregulated, 916 circRNAs were upregulated while 480 were downregulated, 45 miRNAs were upregulated while none were downregulated, and 591 mRNAs were upregulated while 108 were downregulated (*p* ≤ 0.05, and fold change ≥ 2). Gene Ontology (GO) analysis and Kyoto Encyclopedia of Genes and Genomes (KEGG) pathway analysis revealed the biological functions of differentially expressed RNAs. Meanwhile, the RNA networks displayed the regulatory relationship between dysregulated RNAs. Finally, RT‐qPCR validated that the expression of circ‐0033861, circ‐0043273, and circ‐0011959 was upregulated in the plasma exosome of patients with LUAD compared to healthy controls (*p* = 0.0327, *p* = 0.0002, *p* = 0.0437, respectively).

**Conclusion:**

This study proposed a newly discovered ncRNA–miRNA–mRNA/circRNA–miRNA–mRNA ceRNA network and identified that the expression of circulating circ‐0033861, circ‐0043273, and circ‐0011959 was up‐regulated in the plasma exosomes of patients with LUAD, offering valuable insights for exploring the potential function of exosomal noncoding RNA and identifying potential biomarkers for LUAD.

## Graphical abstract

A total of 1016 lncRNAs, 1396 circRNAs, 45 miRNAs, and 699 mRNAs in plasma exosome were differentially expressed in lung adenocarcinoma patients compared with healthy controls using ceRNA microarray, miRNA sequencing. lncRNA‐ and circRNA‐based ceRNA network in plasma exosome from patients with lung adenocarcinoma evaluated the regulatory relationship between dysregulated RNAs. RT‐qPCR verified that circ‐0033861, circ‐0043273, and circ‐0011959 were upregulated in plasma exosome of patients with lung adrnocarcinoma.

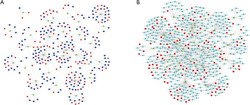

## 1 Introduction

As is well documented, lung cancer is the leading cause of global cancer‐related morbidity and mortality. Non‐small cell lung cancer (NSCLC) accounts for approximately 85% of lung cancer cases, with lung adenocarcinoma (LUAD) being its primary histological subtype. Notably, the incidence of LUAD has progressively increased in recent years [1]. Despite advances in diagnostic and therapeutic methods for lung cancer, the average 5‐year survival rate of patients with LUAD remains below 20% [2, 3, 4]. Given that patients with early‐stage LUAD are mostly asymptomatic, they are typically diagnosed at advanced stages [2, 4, 5, 6]. Thus, there is a pressing need to develop a more accurate diagnostic method to enhance diagnostic accuracy and prognosis.

Competing endogenous RNA (ceRNA), including long noncoding RNAs (lncRNAs), circular RNAs (circRNAs), and pseudogene transcripts, can regulate messenger RNA (mRNA) expression by competitively binding to microRNA (miRNA). These transcripts, also referred to as “RNA sponges”, compete for the same microRNA response elements (MREs) and mutually regulate each other [7, 8]. To date, they have been detected in lung cancer and have been recognized for their crucial role in lung cancer progression [9, 10, 11].

Exosomes are a subset of extracellular vesicles with lipid bilayer structures secreted by various cells. They contain a variety of specific proteins, nucleic acids, and lipids, participating in intercellular communication and play an instrumental role in numerous physiological and pathological processes, including cancer. According to earlier studies, exosomes are rich in noncoding RNAs (ncRNAs), including lncRNAs, circRNAs, as well as miRNAs, which facilitate intracellular communication by transferring exosomes [12, 13, 14, 15].

In recent years, advances in bioinformatics technology have led to the discovery of a large number of lncRNAs and circRNAs with regulatory abilities in various cancers, including lung cancer [2, 16, 17, 18, 19, 20, 21]. Previous studies have reported that lncRNAs are involved in cell proliferation, differentiation, apoptosis, and metastasis through complex regulatory networks composed of miRNAs, mRNAs, and proteins [22, 23]. Furthermore, these lncRNAs can be delivered to target cells or organs through exosomes [24]. The expression level of exosomal lnc‐MLETA1 is positively correlated with the progression of lung cancer. Mechanistically, lnc‐MLETA1 interacts with miR‐186‐5p and miR‐497‐5p to upregulate EGFR and IGF1R expression, thereby promoting lung cancer proliferation [25]. The exosomal lncRNA Mir100hg derived from LUAD could be delivered by exosomes to non‐stemness cancer cells, enhancing their metastatic capability by targeting miR‐15a‐5p and miR‐31‐5P [15]. Unlike linear RNAs, circRNAs are covalent closed loops without a 5′ terminal cap and a 3′ terminal poly(A) tail, a structure that renders circRNAs more stable than linear RNA [13, 22, 23]. circRNAs can also act as ceRNAs by sponging miRNA. For instance, circCCDC134 sponges miR‐625‐5p to regulate target mRNAs [26]. Exosomal lncRNAs, circRNAs, and miRNAs have served as potential biomarkers for the diagnosis and prediction prognosis of lung cancer [27, 28, 29]. Plasmatic lncRNA related to chemotherapy resistance in LUAD (lncCRLA) has been identified as a predictive circulating biomarker for the early diagnosis of preinvasive lesions in patients with LUAD [28]. At the same time, exosomal circZNF451 is associated with a shorter overall survival in patients with lung cancer [30].

However, research on the role of exosomal lncRNAs and circRNAs in the occurrence and development of LUAD is scarce. Moreover, the differential expression profiles of lncRNAs, circRNAs, miRNAs, and mRNAs in LUAD exosomes are underexplored. Therefore, this study adopted microarray and RNA sequencing technology to elucidate the differential expression profiles of lncRNAs, circRNAs, miRNAs, and mRNAs in plasma‐derived exosomes of patients with LUAD to predict possible biological functions in circulating exosomes and reveal new mechanisms of lung adenocarcinoma progression by constructing a ceRNA regulatory network, as well as to identify potential diagnostic biomarkers for LUAD.

## 2 Materials and Methods

### 2.1 Patients and Samples

A total of 77 patients with LUAD admitted to Zhoushan Hospital, Zhejiang Province, from 2017 and 2020 were enrolled. Among them, nine were included for microarray screening and 68 for validation. Patients were pathologically diagnosed with LUAD by two senior pathologists. None of the patients underwent postoperative chemotherapy or radiotherapy. Plasma samples of patients with lung adenocarcinoma were collected before surgery, separated from whole blood in 2 h, and stored at −80 °C. Three sex‐ and gender‐matched healthy controls were included for microarray screening and 45 for validation. Detailed clinicopathological characteristics of patients are presented in Table 1. This study was approved by the Ethical Review Committee of Zhoushan Hospital, and informed consent was obtained from all participants or their families.

**TABLE 1:** Clinicopathological characteristics of nine patients with lung adenocarcinoma for ceRNA and 68 patients for RT‐qPCR. Data are expressed as numbers and percentages.

Characteristic	*n* = 9	*n* = 68
Age (years)	63.2 ± 2.68	61.1 ± 1.42
≤ 60	2(22.2)	32 (47.1)
> 60	7(77.8)	36 (52.9)
Gender		
Male	6(66.7)	22 (32.4)
Female	3(33.3)	46 (67.6)
Pathologic T		
T1	6(66.7)	63 (92.6)
T2	3 (33.3)	5 (7.4)
Predominant subtype		
MIA	0	19 (27.9)
Lepidic‐predominant IAC	0	17 (25.0)
Papillary‐predominant IAC	4 (44.5)	18 (26.5)
Acinar‐predominant IAC	3 (33.3)	11 (16.2)
Cribriform‐predominant IAC	1 (11.1)	1 (1.5)
Solid‐predominant IAC	1 (11.1)	2 (2.9)
Pathologic N		
pN0	4 (44.4)	58 (85.3)
pN1 or 2	5 (55.6)	10 (14.7)
Pathologic stage		
IA	4 (44.4)	55 (80.9)
IB	0	3 (4.4)
IIA	0	7 (10.3)
IIIA	5 (55.6)	3 (4.4)

Abbreviations: IAC, invasive adenocarcinoma; MIA, minimally invasive adenocarcinoma.

### 2.2 Exosome Isolation and Identification and RNA Extraction and Purification

Exosomes were isolated and extracted via ultracentrifugation and visualized under a transmission electron microscope. Exosome size was determined through nanoparticle size analysis, and exosome‐specific surface proteins were detected using fluorescence labeling and a Flow NanoAnalyzer (NanoFCM). Total RNA was extracted using TRIzol reagent (Invitrogen, Thermo Fisher Scientific) and quantified using a NanoDrop ND‐2000 (Thermo Scientific), while RNA integrity was assessed using Agilent Bioanalyzer 2100 (Agilent Technologies). Total RNA was purified using the QIAGEN RNeasy Kit.

### 2.3 Microarray Assay

Purified total RNA (250 ng) was used for labeling and amplification. To begin, an Affinity Script‐RT Kit and random primers were used to reverse‐transcribe RNA into the first strand of complementary DNA (cDNA). Next, an antisense promoter was used to generate the second strand of cDNA, followed by the introduction of T7 RNA polymerase. cRNA was generated through the amplification of the second strand of cDNA. Subsequently, fluorescent dye cyanine‐3‐CTP (Cy3) was used for labeling and purification using a QIAGEN RNeasy Kit. Finally, rolling hybridization was performed at 65 °C for 17 h, and the original image was scanned using an Agilent Scanner G5761A (Agilent Technologies) after elution. Feature extraction software (version 12.0.3.1, Agilent Technologies) was employed to process the original image and extract raw data. Following this, GeneSpring software (version 14.8, Agilent Technologies) was utilized for quantile standardization and subsequent processing. The standardized data were filtered, ensuring that each set of samples for comparison contained at least one set of probes labeled with 100% detection for further analysis. The experiment was repeated in triplicate. The *p* value of the *t‐*test was used for differential gene screening, with the screening criteria comprising a *p* value ≤ 0.05 or a fold change ≥ 2.

### 2.4 Differential Expression Gene Screening and Hierarchical Clustering Analysis

*p* value and fold change value were used to identify differentially expressed genes using the screening criteria of *p* value ≤ 0.05 and fold change value of upregulation or downregulation ≥ 2. A volcano map and heatmap were plotted to visualize the overall distribution of differentially expressed genes. In order to display the expression profiles of lncRNAs, circRNAs, miRNAs, and mRNAs between the two groups, TMEV software was used to conduct hierarchical cluster analysis according to the expression patterns of genes in different samples.

### 2.5 Gene Ontology (GO) and Kyoto Encyclopedia of Genes and Genomes (KEGG) Pathway Enrichment Analyses

GO analysis was conducted to predict the biological processes (BP), cellular components (CC), and molecular functions (MF) of genes in the plasma exosome of patients with LUAD. Meanwhile, KEGG pathway analysis was carried out to determine the involvement of coexpressed genes in biological pathways. Hyper geometric tests were used to identify significantly enriched GO items among dysregulated genes compared with the whole genomic background.

### 2.6 LncRNA‐miRNA‐mRNA/circRNA‐miRNA‐mRNA ceRNA Networks

On the basis of lncRNA–miRNA–mRNA/circRNA–miRNA–mRNA interaction pairs predicted by miRanda and TargetScan software, the relationships between the three RNAs were established to construct lncRNA–miRNA–mRNA and circRNA–miRNA–mRNA coexpression networks, which were visualized using Cytoscape software.

### 2.7 Real‐Time Fluorescence Quantitative Reverse Transcription Polymerase Chain Reaction (RT‐qPCR)

RT‐qPCR was performed to validate the circRNA expression profiles acquired from the microarray data. For circRNAs, 20 U/mL of ribonuclease R (RNase R) was added in RNA to degrade the linear RNA. The cDNA was used as the template in Real‐Time PCR Master Mixes (TaKaRa), with 40 cycles performed in the real‐time PCR system (TaKaRa) and each cycle consisting of denaturation at 95 °C for 5 s and extension at 60 °C for 60 s. GAPDH was used as an endogenous control to normalize each sample. Primers for RT‐qPCR were as follows: circ‐0033861, forward: 5′‐CTTCTTGGTG GCAGCAGCTACAG‐3′, reverse: 5′‐GAGAGGACAGAGGAGTGGATGAGAC‐3′; circ‐0043273, forward: 5′‐TGTTGAGACGGGAGTTTCGCTATTG‐3′, reverse: 5′‐GAGGCAGGAGAATCGCTTGAACC‐3′; and circ‐0011959, forward: 5′‐GAGGCA GGAGAATCGCTTGAACC‐3′, reverse: 5′‐TTGGAGATGGAGTTTCGCTCTTG TC‐3′, GAPDH, 5′‐GACCTGACCTGCCGTCTA‐3′, 5′‐AGGAGTGGGTGTCGCTG T‐3′. The 2‐△△Ct method was used to determine the fold change in gene expression in LUAD samples relative to normal samples.

### 2.8 Statistical Analysis

An unpaired *t‐*test was applied to compare the *p*‐values of differential gene screening in the microarray analysis, with screening criteria of *p* value ≤ 0.05 and fold change ≥ 2. Regarding RT‐qPCR, the unpaired *t‐*test was conducted to compare RNA expression levels in patients with LUAD and healthy controls. Statistical analyses were performed using GraphPad Prism9 software (GraphPad Software). A *p* value ≤ 0.05 was considered statistically significant.

## 3 Results

### 3.1 Identification and Characterization of Isolated Plasma Exosomes

**FIGURE 1:**
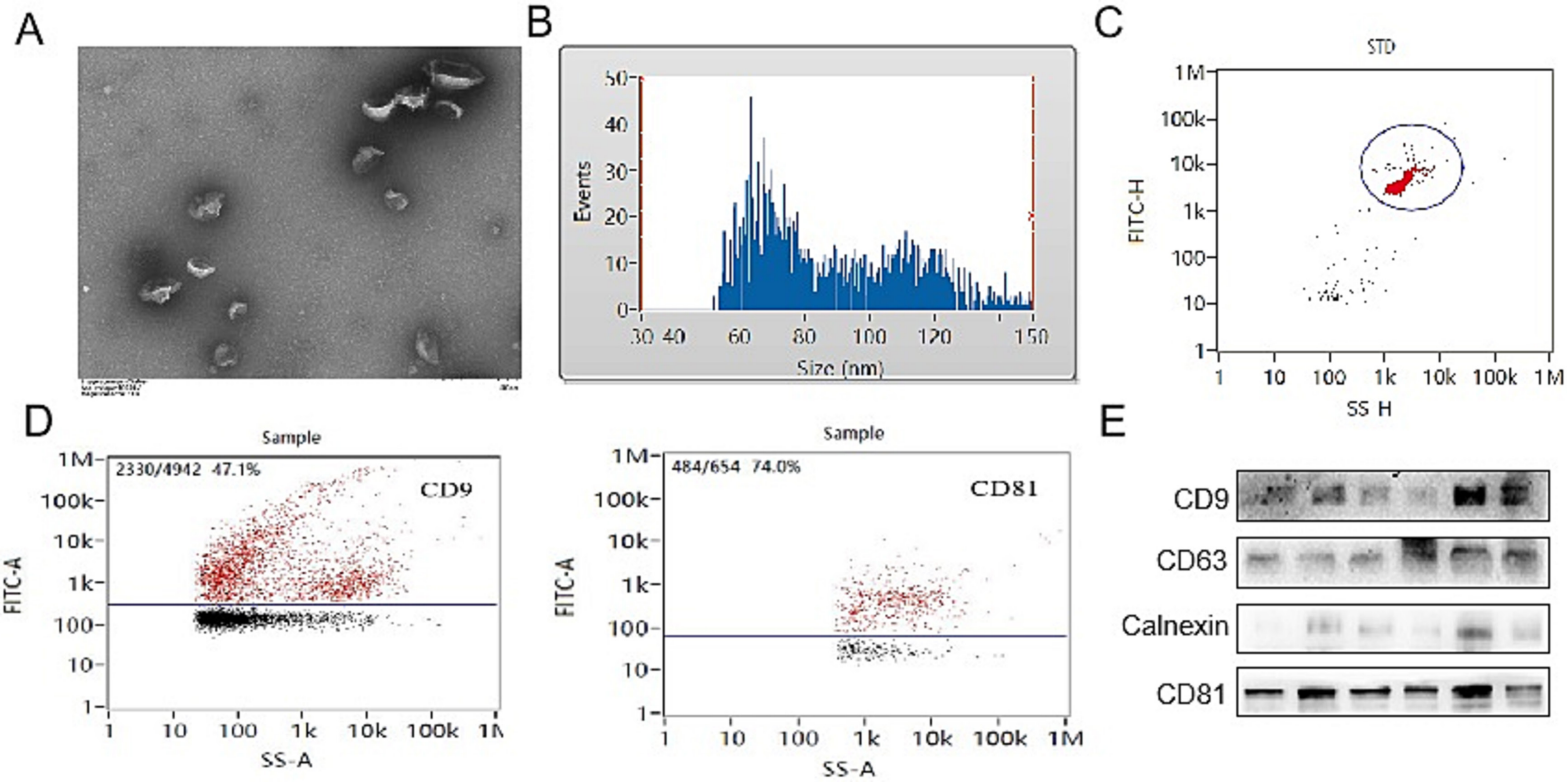
Identification of exosomes isolated from the plasma of patients with LUAD. (A) Representative image of exosomes observed under transmission electron microscopy. (B) Particle size distribution of exosomes, as measured with nanoparticle tracking analysis. (C) Exosomes were detected using flow cytometry. (D) Flow cytometry analysis of exosome‐related proteins CD9 and CD81 levels. (E) The expression levels of the exosome markers CD9, CD63, CD81, and calnexin were detected via Western blot analysis. LUAD, lung adenocarcinoma.

Exosomes were isolated from the plasma of patients with LUAD and healthy donors. Transmission electron microscopy, nanoparticle size analysis, and nanoflow cytometry were performed to observe the characteristics of exosomes. The results displayed that the separated vesicles were spherical and cup‐ and disc‐shaped (Figure 1A). Moreover, nanoparticle size analysis revealed that the average particle size of the obtained vesicles was 89.06 ± 24.26 nm (Figure 1B). The isolated vesicles were also detected via nanoflow cytometry, uncovering positive rates of exosomal markers CD9 and CD81 at 47.1% and 74.0%, respectively (Figure 1C,D). Western blot analysis detected the presence of the exosome related protein markers CD9, CD63, and CD81 in the plasma exosome of patients with LUAD (Figure 1E). Taken together, the aforementioned results indicated that the vesicles isolated from plasma were indeed exosomes.

### 3.2 Microarray Results of Differentially Expressed Plasma Exosomal lncRNA, circRNA, miRNA, and mRNA Profiles of Patients With LUAD

**FIGURE 2:**
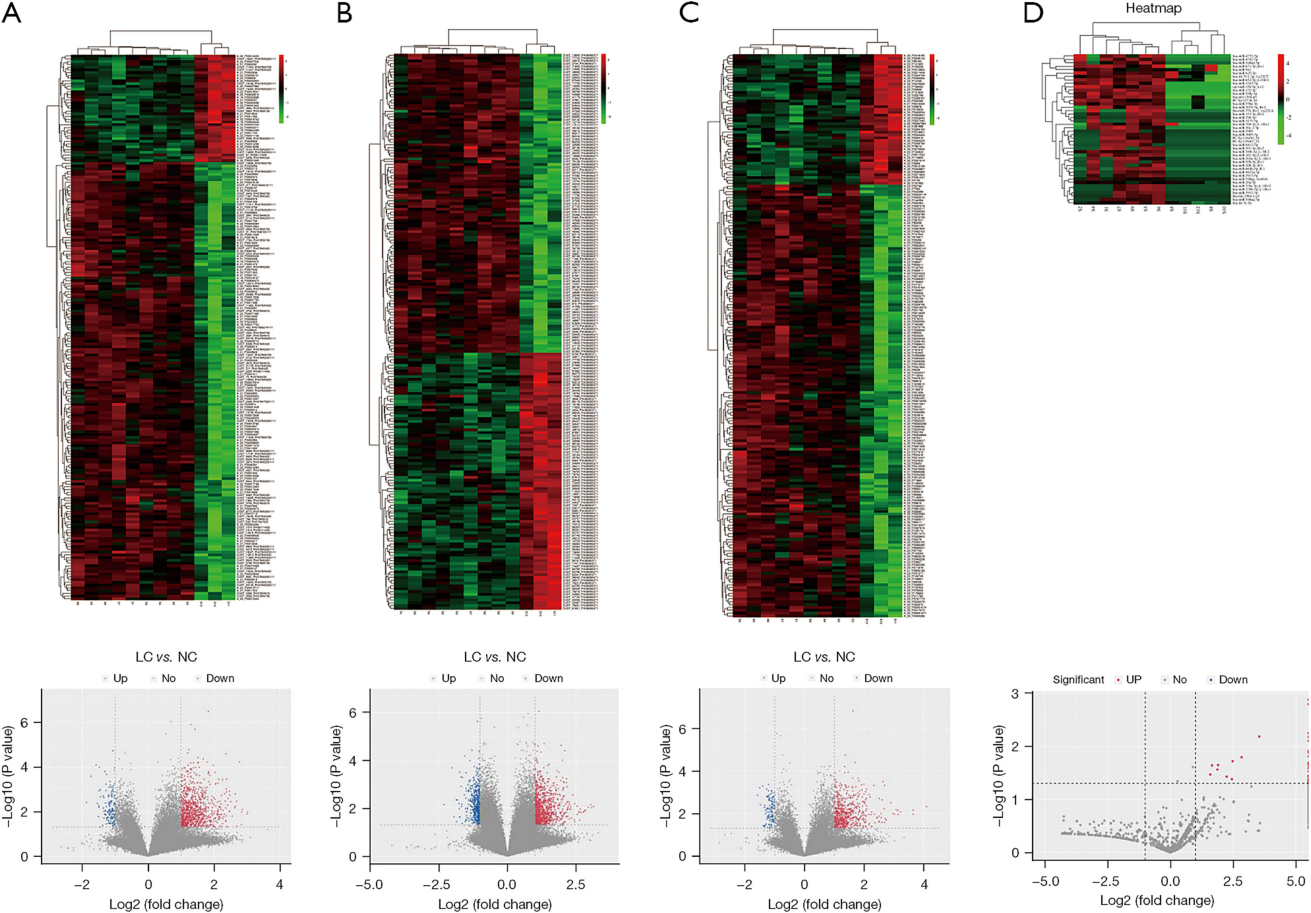
Differentially expressed plasma exosomal lncRNA, circRNA, miRNA, and mRNA profiles between patients with lung adenocarcinoma and normal controls identified via microarray analysis. Heatmaps and volcano plots of the differentially expressed (A) mRNAs, (B) lncRNAs, (C) circRNAs, and (D) miRNAs. Red represents up‐regulated genes, and green represents down‐regulated genes. lncRNA, long noncoding RNA; circRNA, circular RNA; miRNA, microRNA; mRNA, messenger RNA.

**FIGURE 3:**
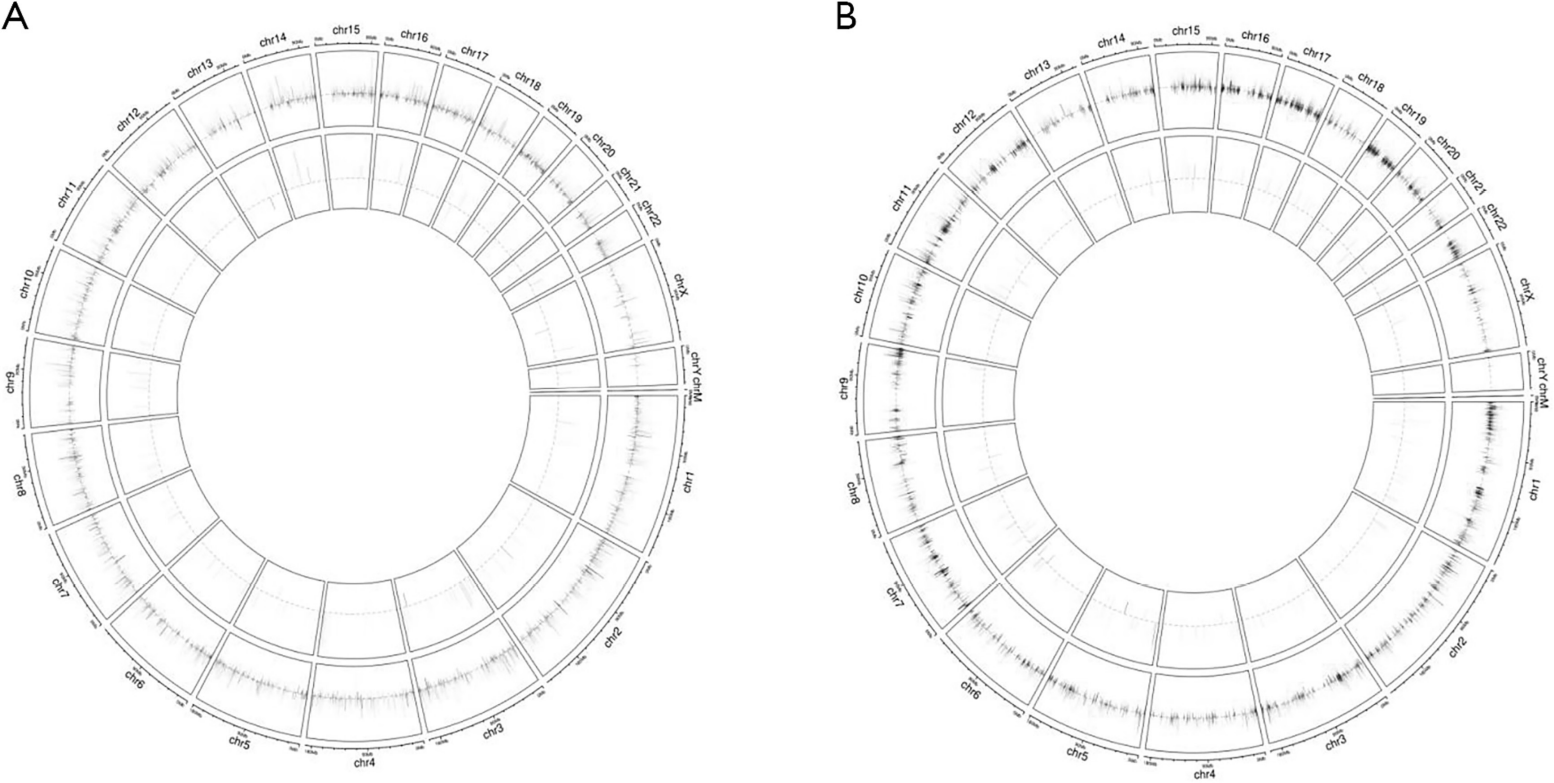
Acircos diagram illustrating the distribution of differentially expressed (A) lncRNAs and (B) circRNAs identified in plasma exosomes derived from patients with LUAD and matched healthy controls. lncRNA, long noncoding RNA, circRNA, circular RNA. LUAD, lung adenocarcinoma.

Microarray and RNA sequencing results identified 1016 lncRNAs, 1396 circRNAs, 45 miRNAs, and 699 mRNAs that were significantly dysregulated in the plasma exosomes of patients with LUAD compared with healthy controls. Among them, 881 lncRNAs were upregulated whereas 135 were downregulated, 916 circRNAs were upregulated whereas 480 were downregulated, 45 miRNAs were upregulated whereas none were downregulated, and 591 mRNAs were upregulated whereas 108 were downregulated (*p* ≤ 0.05 and fold change ≥ 2; Figure 2A–D). Moreover, the volcano data illustrated differences in the expression levels of lncRNAs, circRNAs, miRNAs, and mRNAs between the plasma exosomes of patients with LUAD and those of healthy controls (Figure 2A–D). The circos diagram in Figure 3A–B illustrates the distribution of the differentially regulated lncRNAs and circRNAs across chromosomes in the plasma exosomes of patients with LUAD. As anticipated, they were distributed on most chromosomes. These results collectively suggest that the expression levels of lncRNAs, circRNAs, miRNAs, and mRNAs in the plasma exosomes of patients with LUAD were markedly different from those of healthy individuals.

### 3.3 GO and KEGG Enrichment Analysis of Plasma Exosomal lncRNA and circRNA Coexpression With mRNA

**FIGURE 4:**
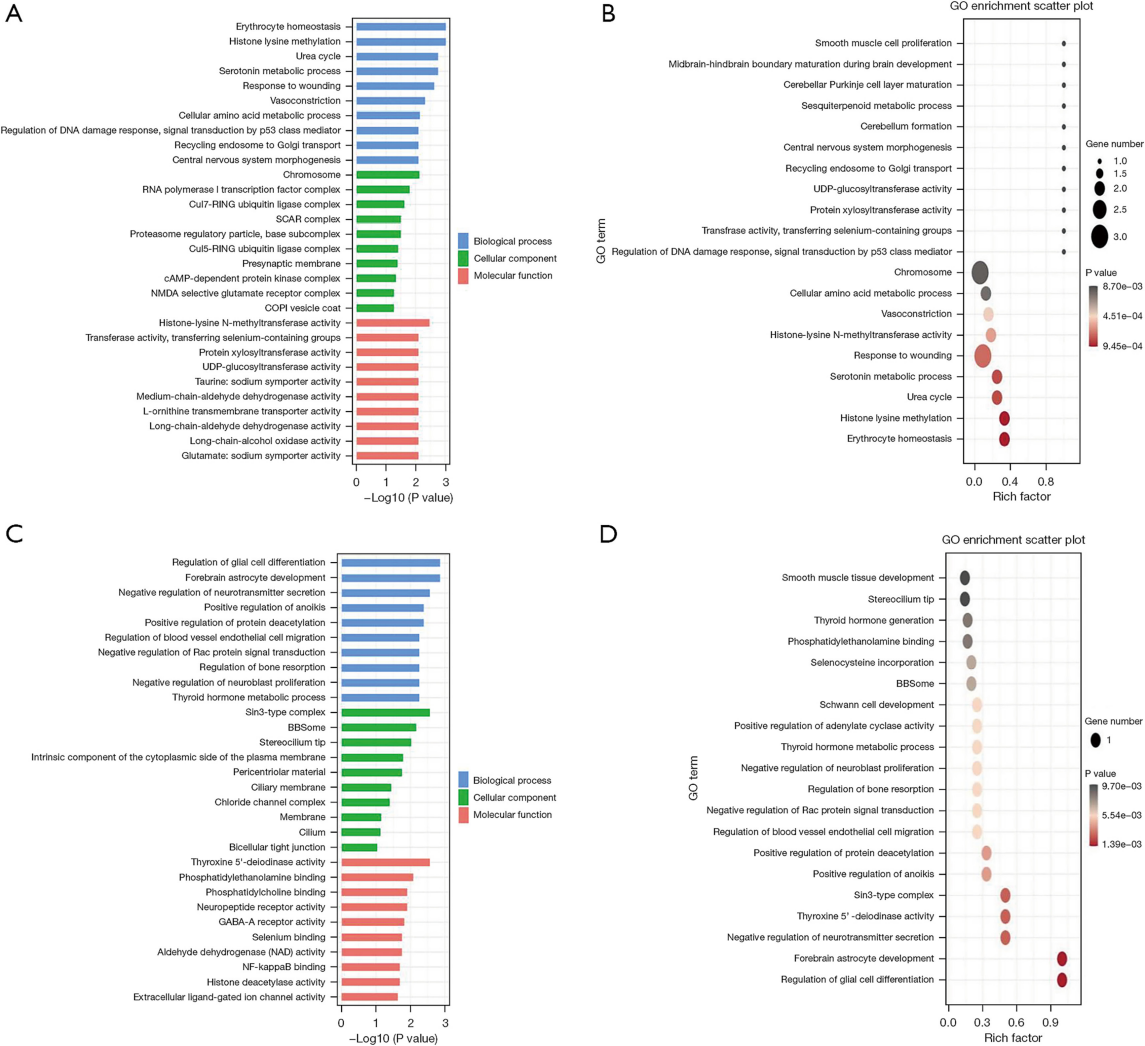
GO analysis of the functions of plasma exosomal lncRNAs isolated from the plasma of patients with lung adenocarcinoma. GO annotation for upregulated lncRNAs and coexpressed mRNAs. (A) histogram and (B) scatter plot. (C) Histogram and (D) scatter plot for the downregulated lncRNAs and coexpressed mRNAs with top enrichment scores across the domains of BP, CC, and MF. lncRNA, long noncoding RNA; circRNA, circular RNA; GO, Gene Ontology; BP, biological process; CC, cellular component; MF, molecular function.

**FIGURE 5:**
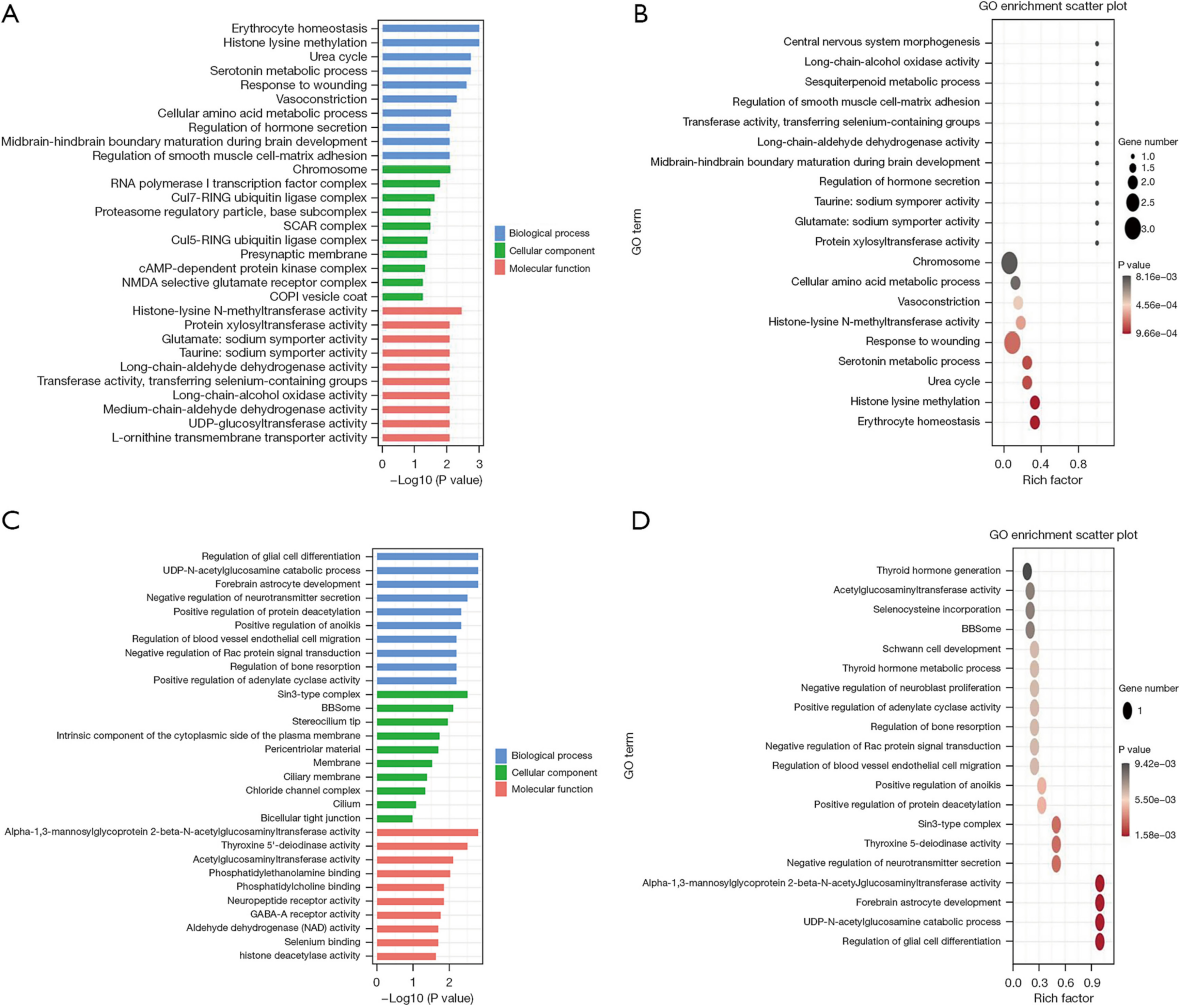
GO analysis of exosomal circRNAd derived from the plasma of patients with lung adenocarcinoma. GO annotation for upregulated circRNAs and coexpressed mRNAs. (A) histogram and (B) scatter plot. (C) Histogram and (D) scatter plot for the downregulated circRNAs and coexpressed mRNAs with top enrichment scores across the domains of BP, CC, and MF. lncRNA, long noncoding RNA; circRNA, circular RNA; GO, Gene Ontology; BP, biological process; CC, cellular component; MF, molecular function.

**FIGURE 6:**
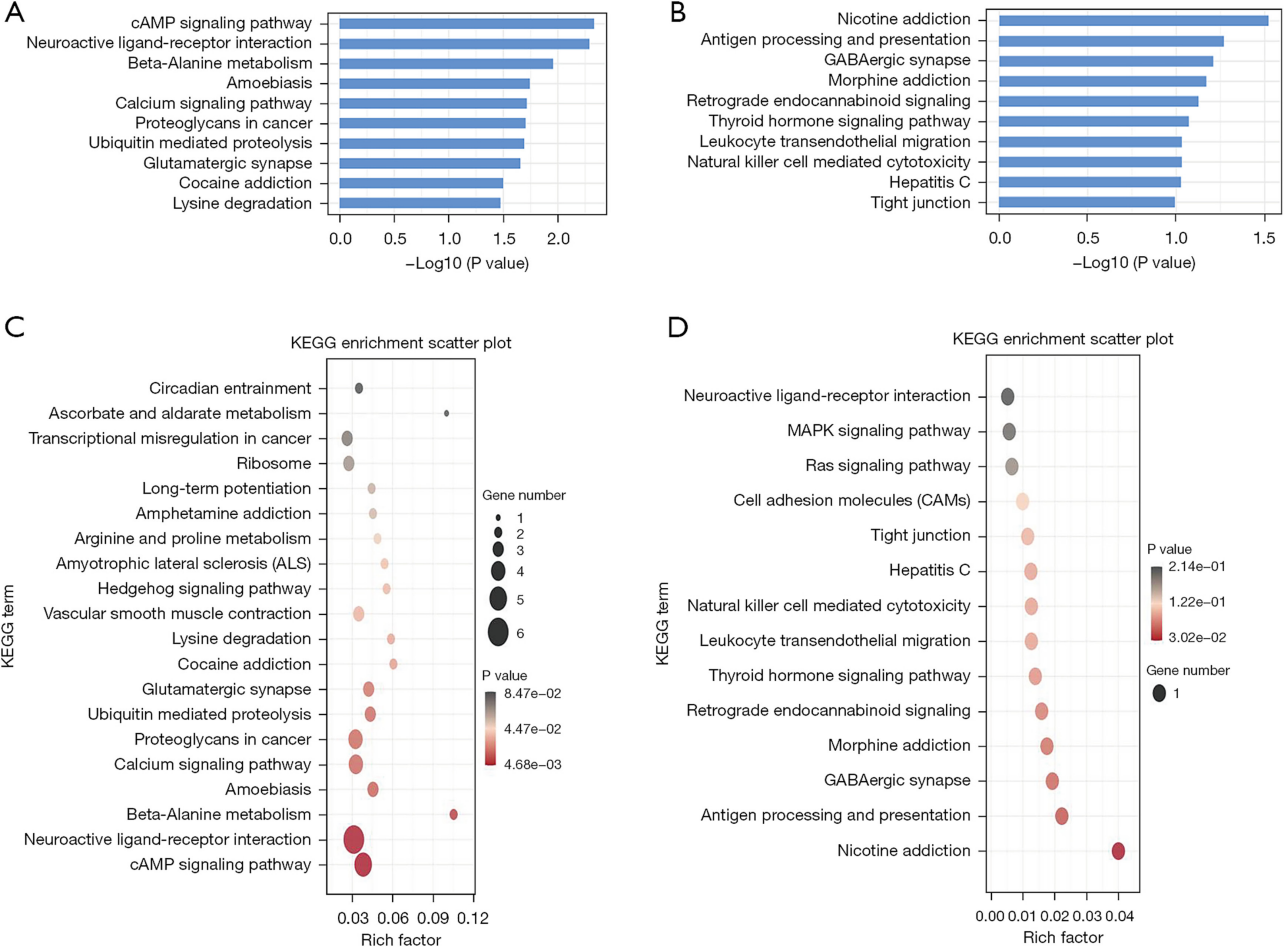
KEGG analysis of the functions of exosomal lncRNA isolated from the plasma of patients with lung adenocarcinoma. (A) Histogram and (B) scatter plot depicting upregulated lncRNAs and coexpressed mRNAs. (C) Histogram and (D) illustrating downregulated lncRNAs and coexpressed mRNAs with the highest enrichment scores. lncRNA, long noncoding RNA; circRNA, circular RNA, KEGG, Kyoto Encyclopedia of Genes and Genomes.

**FIGURE 7:**
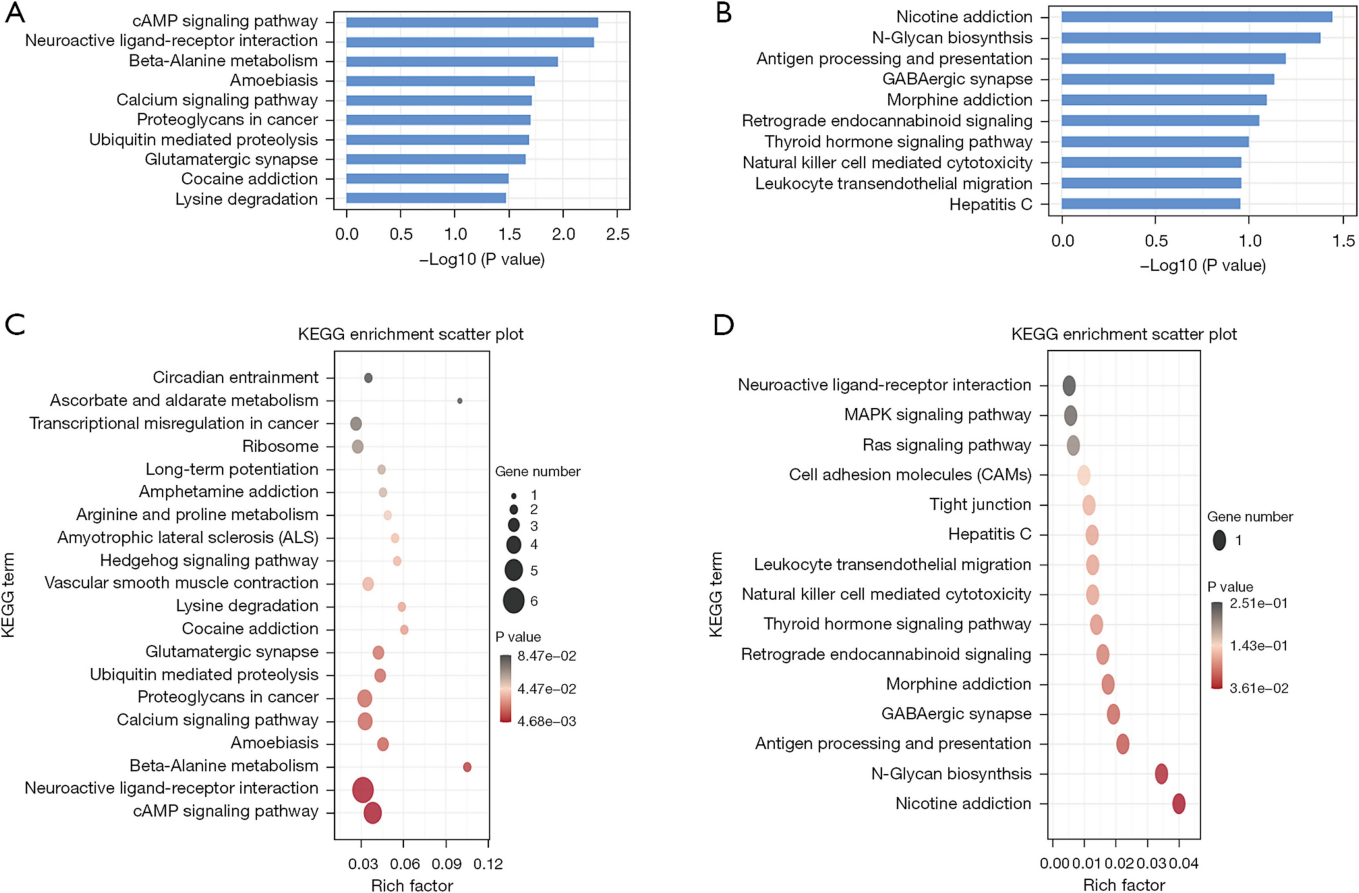
KEGG analysis of pathways of exosomal circRNA isolated from the plasma of patients with lung adenocarcinoma. (A) Histogram and (B) scatter plot presenting upregulated circRNAs and coexpressed mRNAs. (C) Histogram and (D) scatter plot delineating downregulated circRNAs and coexpressed mRNAs with the highest enrichment scores. lncRNA, long noncoding RNA; circRNA, circular RNA; KEGG, Kyoto Encyclopedia of Genes and Genomes.

To elucidate the functions of the differentially expressed lncRNAs and circRNAs, GO enrichment analysis and KEGG pathway analysis were conducted on the differentially expressed lncRNAs and circRNAs co‐expressed with mRNAs between patients with LUAD and normal controls. The top 10 GO terms with the most significant changes were classified into BP, CC, or MF. Compared with mRNAs in normal controls, those upregulated by lncRNAs in patients with LUAD were associated with erythrocyte homeostasis and histone lysine methylation. Moreover, chromosome and histone‐lysine N‐methyltransferase activity were the most abundant classes in the CC and MF categories, respectively (Figure 4A–B). Conversely, downregulated transcripts were associated with BPs such as the regulation of glial cell differentiation and forebrain astrocyte development, whereas sin3‐type complex and thyroxine 5′‐deiodinase activity were the most abundant classes in the CC and MF categories, respectively (Figure 4C–D). Likewise, the BP of mRNAs upregulated by circRNAs was associated with erythrocyte homeostasis and histone lysine methylation. In terms of CC and MF, chromosome and histone‐lysine N‐methyltransferase activity were also the most abundant classes in circRNA‐mRNA coexpression (Figure 5A–B). Finally, the BPs associated with mRNAs downregulated by circRNAs included the deregulation of glial cell differentiation and the UDP‐N‐acetylglucosamine catabolic process. The highest enrichment scores for CCs and MFs were sin3‐type complex and alpha‐1,3‐mannosyl glycoprotein 2‐beta‐N‐acetylglucosaminyl transferase activity (Figure 5C–D). KEGG pathway enrichment analysis identified the top 10 pathways associated with upregulated and downregulated mRNAs coexpressed with lncRNAs and circRNAs. For lncRNAs, the cAMP signaling pathway and nicotine addiction were the most enriched pathways of dysregulated genes (Figure 6A–D). For circRNAs, the most enriched KEGG pathways for coexpressed upregulated and downregulated mRNAs were nicotine addiction and cAMP signaling pathways, respectively (Figure 7A–D).

### 3.4 Construction of the ceRNA Network

**FIGURE 8:**
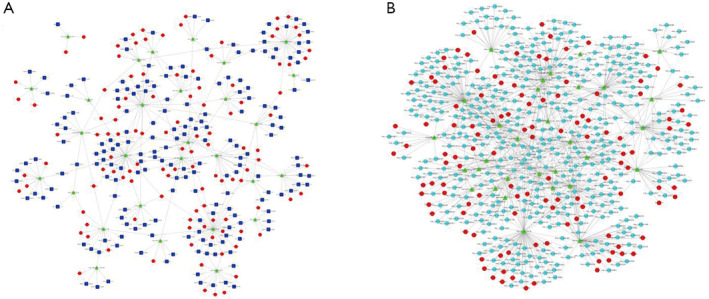
Competing endogenous RNA network in LUAD. The competing endogenous RNA network was based on lncRNA‐miRNA, circRNA‐miRNA, and miRNA‐mRNA interactions. In this network, each point represents a gene, and the straight lines denote interactions between genes (*p* ≤ 0.05 and fold change ≥ 2). LUAD, lung adenocarcinoma; lncRNA, long noncoding RNA; circRNA, circular RNA; miRNA, microRNA.

According to the ceRNA hypothesis, ceRNAs can compete for the same MRE to exert mutual regulation. lncRNAs and circRNAs regulate gene expression by interacting with miRNAs and act as effective miRNA sponges, thereby influencing tumor progression. Thus, a lncRNA/circRNA–miRNA–mRNA ceRNA network was constructed. A total of 189 lncRNAs, 29 miRNAs, and 114 mRNAs were used to construct the lncRNA‐mediated coexpression network (Figure 8A), while 424 circRNAs, 31 miRNAs, and 117 mRNAs were selected to construct the circRNA‐mediated network, as depicted in Figure 8B, wherein the size of the graph represents the magnitude of the fold‐change, with a larger size indicating a greater fold change. The network contained 363 and 780 edges for the lncRNA‐ and circRNA‐mediated networks, respectively (Table 2).

**TABLE 2:** The nodes and edges in the LUAD dysregulated ceRNA network.

lncRNA‐mediated ceRNA network		circRNA‐mediated ceRNA network	
lncRNAs	189	circRNAs	424
miRNAs	29	miRNAs	31
mRNAs	114	mRNAs	117
Edges	363	edges	780

Abbreviations: lncRNA, long noncoding RNA; LUAD, lung adenocarcinoma; miRNA, microRNA; mRNA, messenger RNA.

### 3.5 Validation of Deregulated Plasma Exosomal circRNAs With RT‐qPCR

**FIGURE 9:**
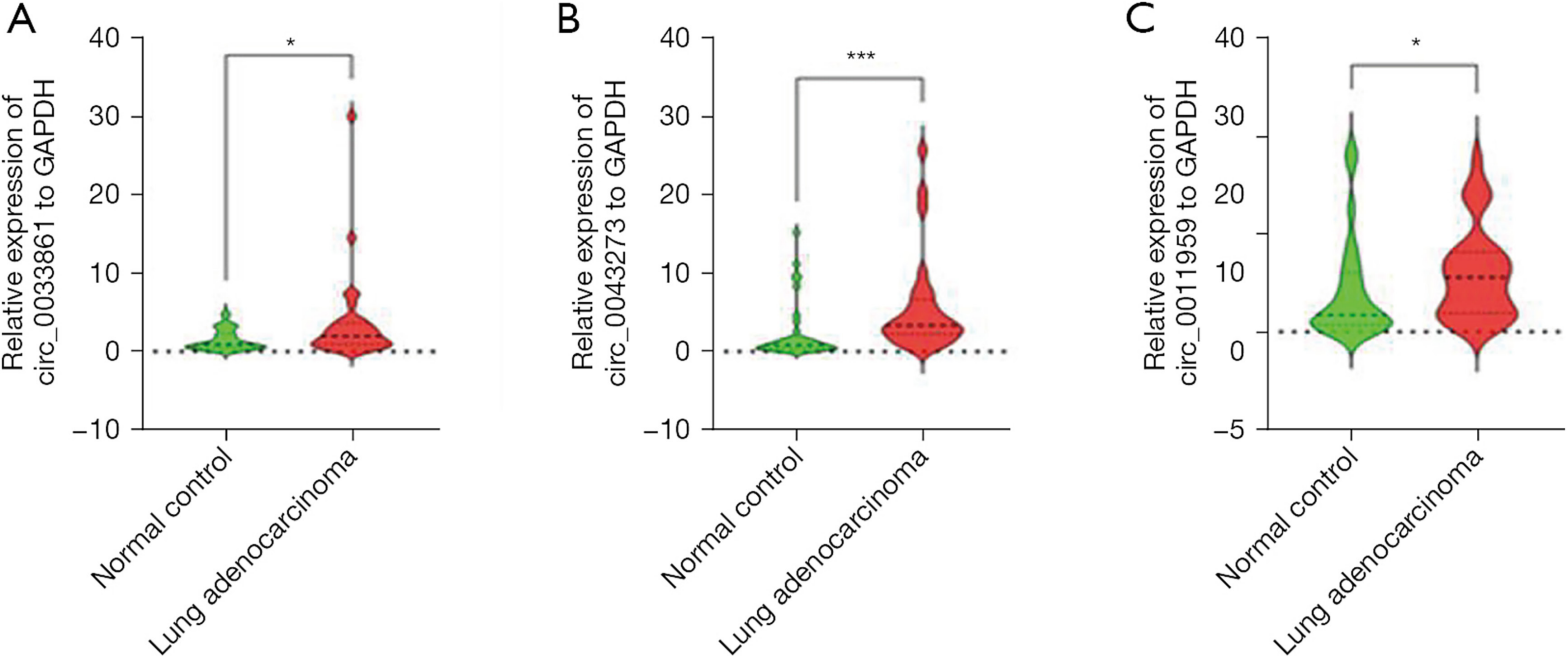
Validation of circRNA expression levels in the plasma exosomes of patients with lung adenocarcinoma. (A) Relative expression levels of circ_0033861, (B) circ_0043273, and (C) circ_0011959 in the plasma exosomes of patients with lung adenocarcinoma compared to normal controls. Data are expressed as mean ± standard deviation. * * * *p* ≤ 0.01, * *p* ≤ 0.05.

To further confirm the data of microarray, RT‐qPCR was performed to detect the expression level of circ‐0033861, circ‐0043273, and circ‐0011959 in plasma exosomes of patients with LUAD and normal controls. The results indicated that the expression levels of circ‐0033861, circ‐0043273, and circ‐0011959 were upregulated in the plasma exosomes of patients with LUAD compared to normal controls (*p* = 0.0327, *p* = 0.0002, *p* = 0.0437, respectively; Figure 9A–C), consistent with the microarray data.

## 4 Discussion

Existing studies have highlighted the role of the ceRNA regulatory network in cancers. On the other hand, ceRNA in exosomes extracted from the plasma of patients with LUAD remains to be elucidated. Our study revealed specific expression levels of lncRNAs, circRNAs, miRNAs, and mRNAs extracted from the plasma exosomes of patients with LUAD and healthy controls. GO analyses unveiled relatively frequent pathway alterations, such as the histone lysine methylation of lncRNAs and circRNAs. Histone lysine methylation plays a vital role in cancer progression, including lung cancer, implying that exosomal lncRNA or circRNA alterations are correlated with tumorigenesis [31]. Moreover, our findings demonstrated that lncRNA and circRNA share identical GO pathways, suggesting overlapping enrichment targets for mRNA [32].

Furthermore, our results suggested that the enrichment pathways in lncRNA‐mRNA coexpression alterations were similar to those in circRNA‐mRNA, demonstrating that the target mRNAs of exosomal lncRNAs and circRNAs may share the same MREs and act as a type of ceRNA. The dysregulated exosomal lncRNAs and circRNAs in LUAD participate in tumor regulatory pathways through exosomes and ultimately affect the progression of LUAD. Among the pathways identified by KEGG, our results revealed that the cAMP signaling pathway had the highest enrichment fraction in the upregulation of lncRNA/circRNA‐mRNA. Previous studies have reported that the cAMP signaling system mediates cellular functions, including cell metabolism, proliferation, apoptosis, autophagy, drug resistance, invasion, and metastasis, playing an essential role in the pathogenesis of lung cancer [33, 34, 35, 36, 37, 38, 39]. According to an earlier study, the deletion of lncRNA NEAT1 contributed to epithelial regeneration by activating the cAMP signaling pathway [40]. Conversely, cAMP up‐regulated the expression of lnc473 through STAT3 phosphorylation [41]. Moreover, circLARP1B upregulated phosphodiesterase 4C to repress cAMP signaling [42]. The mechanism underlying the relationships between lncRNAs/circRNAs and the cAMP signaling pathway remains underexplored, warranting further studies to elucidate their role in tumor progression.

The constructed lncRNA‐miRNA‐mRNA network based on the circulating exosomes comprised 189 lncRNAs, 29 miRNAs, and 114 mRNAs. Among them, exosomal lnc‐AKAP14‐1:2 was the most upregulated lncRNA. Mounting evidence suggests that exosomal lncRNAs are potential non‐invasive effective biomarkers for the diagnosis and prognosis of lung cancer [11, 43, 44]. A total of 424 circRNAs, 31 miRNAs, and 117 mRNAs were included in the circRNA‐miRNA‐mRNA network. Among them, exosomal circ_0041755 (circRNASEK), circ_0043273 (circTADA2A), and circ_0033861 (circADAM6) were the most upregulated circRNAs and may be regarded as candidate biomarkers for the diagnosis of LUAD. circTADA2A can sponge miR‐520f, miR‐455‐3p, and miR‐129‐5p to compete with target mRNAs to promote tumor proliferation, drug resistance, and carcinogenesis in several types of cancers, including lung cancer [45, 46, 47]. However, circTADA2A binds to miR‐374a‐3p, functioning as a ceRNA to regulate target KLF14 and inhibit tumor proliferation in colorectal cancer [48]. Nonetheless, its role should be further investigated. Based on the miRNA–mRNA–lncRNA network, lncADAM6 was identified as a ceRNA and might be a potential biomarker for LUAD prognosis. However, studies examining the role of circADAM6 in tumor progression are limited [49].

Circulating exosomal circRNAs have been identified as potential biomarkers for predicting the diagnosis and prognosis of lung cancer owing to their stability and their ability to reflect tumor gene expression [11, 44, 50, 51]. Thus, they were selected to validate the results of the ceRNA network. Our results demonstrated that the expression levels of circ_0043273, circ_0011959, and circ_0033861 were significantly higher in the plasma exosomes of patients with LUAD than those of controls, in agreement with the expression patterns observed in the microarray analysis data. This finding corroborates their potential as informative circulating non‐invasive biomarkers for the diagnosis of LUAD. The role of circulating circRNA in predicting the diagnosis and prognosis of LUAD has been established and validated for future applications.

## 5 Conclusions

Collectively, the current study proposed a novel lncRNA/circRNA–miRNA–mRNA ceRNA regulatory network in circulating exosomes extracted from patients with LUAD, thereby laying a theoretical reference for exploring the potential functions and molecular mechanisms of exosomal RNA in LUAD. In addition, the current study also revealed that circulating levels of exosomal circ_0043273, circ_0011959, and circ_0033861 were higher in patients with LUAD compared with healthy controls, providing novel potential diagnostic biomarkers for the diagnosis of LUAD. However, further prospective studies are necessary to verify the efficacy of exosomal circRNAs in the early diagnosis of LUAD.

## Author Contributions

(I) Conception and design: Wangyu Zhu and Hanbo Le; (II) Administrative support: Hanbo Le; (III) Provision of study materials or patients: Huafeng Zhang, Liwei Tang, and Yongkui Zhang; (IV) Collection and assembly of data: Huafeng Zhang, Liwei Tang, Kexin Fang, Nawa Lin, and Yanyan Huang; (V) Data analysis and interpretation: Wangyu Zhu, Huafeng Zhang, and Liwei Tang; (VI) Manuscript writing: All authors; (VII) Final approval of manuscript: All authors.

## Ethics Statement

All procedures performed in studies involving human participants were in accordance with the ethical standards of the institutional and/or national research committee and with the 1964 Helsinki declaration and its later amendments or comparable ethical standards.

## Conflicts of Interest

The authors declare no conflicts of interest.

## Data Availability

The data in this study can be obtained under reasonable conditions by contacting Dr. Wangyu Zhu (email: zhuwangyu24@sina.cn).

## References

[1] R. L. Siegel , A. N. Giaquinto , and A. Jemal , “Cancer Statistics, 2024,” CA: A Cancer Journal for Clinicians 74 (2024): 12–49. 38230766 10.3322/caac.21820

[2] X. Wang , R. Su , Q. Guo , J. Liu , B. Ruan , and G. Wang , “Competing Endogenous RNA (ceRNA) Hypothetic Model Based on Comprehensive Analysis of Long Non‐Coding RNA Expression in Lung Adenocarcinoma,” PeerJ 2019 (2019): 1–17. 10.7717/peerj.8024 PMC6842565 31720124

[3] S. Xie , Z. Wu , Y. Qi , B. Wu , and X. Zhu , “The Metastasizing Mechanisms of Lung Cancer: Recent Advances and Therapeutic Challenges,” Biomedicine & Pharmacotherapy 138 (2021): 111450. 33690088 10.1016/j.biopha.2021.111450

[4] T. B. Kratzer , P. Bandi , N. D. Freedman , et al., “Lung Cancer Statistics, 2023,” Cancer 130 (2024): 1330–1348. 38279776 10.1002/cncr.35128

[5] F. M. Carozzi , S. Bisanzi , L. Carrozzi , et al., “Multimodal Lung Cancer Screening Using the ITALUNG Biomarker Panel and low Dose Computed Tomography. Results of the ITALUNG Biomarker Study,” International Journal of Cancer 141 (2017): 94–101. 28387927 10.1002/ijc.30727

[6] R. L. Siegel , K. D. Miller , H. E. Fuchs , and A. Jemal , “Cancer Statistics, 2021,” CA: A Cancer Journal for Clinicians 71 (2021): 7–33. 33433946 10.3322/caac.21654

[7] L. Salmena , L. Poliseno , Y. Tay , L. Kats , and P. P. Pandolfi , “A ceRNA Hypothesis: The Rosetta Stone of a Hidden RNA Language?,” Cell 146 (2011): 353–358. 21802130 10.1016/j.cell.2011.07.014 PMC3235919

[8] X. Qi , D. H. Zhang , N. Wu , J. H. Xiao , X. Wang , and W. Ma , “ceRNA in Cancer: Possible Functions and Clinical Implications,” Journal of Medical Genetics 52 (2015): 710–718. 26358722 10.1136/jmedgenet-2015-103334

[9] Z. Ting , Z. Wu , C. Yang , et al., “lncRNA CERS6‐AS1 Upregulates the Expression of ANLN by Sponging miR‐424‐5p to Promote the Progression and Drug Resistance of Lung Adenocarcinoma,” Noncoding RNA Research 9 (2024): 221–235. 10.1016/j.ncrna.2023.11.013 PMC10716711 38094657

[10] Y. Li , X. Hong , J. Zhai , et al., “Novel Circular RNA Circ‐0002727 Regulates miR‐144‐3p/KIF14 Pathway to Promote Lung Adenocarcinoma Progression,” Frontiers in Cell and Development Biology 11 (2023): 1249174. 10.3389/fcell.2023.1249174 PMC10686231 38033864

[11] R. Wang , Y. Xu , L. Tong , X. Zhang , and S. Zhang , “Recent Progress of Exosomal lncRNA/circRNA‐miRNA‐mRNA Axis in Lung Cancer: Implication for Clinical Application,” Frontiers in Molecular Biosciences 11 (2024): 1417306. 39021878 10.3389/fmolb.2024.1417306 PMC11251945

[12] I. Vanni , A. Alama , F. Grossi , M. G. Dal Bello , and S. Coco , “Exosomes: A New Horizon in Lung Cancer,” Drug Discovery Today 22 (2017): 927–936. 28288782 10.1016/j.drudis.2017.03.004

[13] D. Fanale , S. Taverna , A. Russo , and V. Bazan , “Circular RNA in Exosomes,” Advances in Experimental Medicine and Biology 1087 (2018): 109–117. 30259361 10.1007/978-981-13-1426-1_9

[14] Y. Wang , J. Zhang , H. Shi , et al., “M2 Tumor‐Associated Macrophages‐Derived Exosomal MALAT1 Promotes Glycolysis and Gastric Cancer Progression,” Advanced Science (Weinheim) 11 (2024): e2309298. 10.1002/advs.202309298 PMC11199979 38639382

[15] L. Shi , B. Li , Y. Zhang , et al., “Exosomal lncRNA Mir100hg Derived From Cancer Stem Cells Enhance Glycolysis and Promote Metastasis of Lung Adenocarcinoma Through mircroRNA‐15a‐5p/31‐5p,” Cell Communication and Signaling: CCS 21 (2023): 248. 37735657 10.1186/s12964-023-01281-3 PMC10512609

[16] S. Dong , C. Wu , C. Song , B. Qi , L. Liu , and Y. Xu , “Identification of Primary and Metastatic Lung Cancer‐Related lncRNAs and Potential Targeted Drugs Based on ceRNA Network,” Frontiers in Oncology 10 (2021): 1–12. 10.3389/fonc.2020.628930 PMC7886985 33614509

[17] R. Dong , J. Liu , W. Sun , and W. Ping , “Comprehensive Analysis of Aberrantly Expressed Profiles of lncRNAs and miRNAs With Associated ceRNA Network in Lung Adenocarcinoma and Lung Squamous Cell Carcinoma,” Pathology Oncology Research 26 (2020): 1935–1945. 31898160 10.1007/s12253-019-00780-4

[18] X. Wu , Z. Sui , H. Zhang , Y. Wang , and Z. Yu , “Integrated Analysis of lncRNA–Mediated ceRNA Network in Lung Adenocarcinoma,” Frontiers in Oncology 10 (2020): 1–10. 33042838 10.3389/fonc.2020.554759 PMC7523091

[19] F. Chen , C. Huang , Q. Wu , L. Jiang , S. Chen , and L. Chen , “Circular RNAs Expression Profiles in Plasma Exosomes From Early‐Stage Lung Adenocarcinoma and the Potential Biomarkers,” Journal of Cellular Biochemistry 121 (2020): 2525–2533. 31646690 10.1002/jcb.29475

[20] F. Fan , Y. Ping , L. Yang , et al., “Characterization of a Non‐Coding RNA‐Associated ceRNA Network in Metastatic Lung Adenocarcinoma,” Journal of Cellular and Molecular Medicine 24 (2020): 11680–11690. 32860342 10.1111/jcmm.15778 PMC7579711

[21] X. Kong , S. Hu , Y. Yuan , et al., “Analysis of lncRNA, miRNA and mRNA‐Associated ceRNA Networks and Identification of Potential Drug Targets for Drug‐Resistant Non‐Small Cell Lung Cancer,” Journal of Cancer 11 (2020): 3357–3368. 32231742 10.7150/jca.40729 PMC7097957

[22] A. A. Ishola , A. S. La'ah , H. D. Le , et al., “Non‐Coding RNA and Lung Cancer Progression,” Journal of the Chinese Medical Association : JCMA 83 (2020): 8–14. 31770191 10.1097/JCMA.0000000000000225 PMC13047976

[23] J. Beermann , M. T. Piccoli , J. Viereck , and T. Thum , “Non‐coding Rnas in Development and Disease: Background, Mechanisms, and Therapeutic Approaches,” Physiological Reviews 96 (2016): 1297–1325. 27535639 10.1152/physrev.00041.2015

[24] S. Liu , S. Wang , J. Guo , et al., “Crosstalk Among Disulfidptosis‐Related lncRNAs in Lung Adenocarcinoma Reveals a Correlation With Immune Profile and Clinical Prognosis,” Noncoding RNA Research 9 (2024): 772–781. 10.1016/j.ncrna.2024.03.006 PMC10999374 38590434

[25] X. R. Hsu , J. E. Wu , Y. Y. Wu , et al., “Exosomal Long Noncoding RNA MLETA1 Promotes Tumor Progression and Metastasis by Regulating the miR‐186‐5p/EGFR and miR‐497‐5p/IGF1R Axes in non‐small Cell Lung Cancer,” Journal of Experimental & Clinical Cancer Rresearch: CR 42 (2023): 283. 10.1186/s13046-023-02859-y PMC10601119 37880793

[26] Z. Tong , Z. Wang , J. Jiang , C. Tong , and L. Wu , “A Novel Molecular Mechanism Mediated by circCCDC134 Regulates Non‐Small Cell Lung Cancer Progression,” Thoracic Cancer 14 (2023): 1958–1968. 37231545 10.1111/1759-7714.14942 PMC10344740

[27] M. Hashemi , E. M. Khosroshahi , M. K. Chegini , et al., “miRNAs and Exosomal miRNAs in Lung Cancer: New Emerging Players in Tumor Progression and Therapy Response,” Pathology, Research and Practice 251 (2023): 154906. 37939448 10.1016/j.prp.2023.154906

[28] S. Lin , C. He , L. Song , et al., “Exosomal lncCRLA Is Predictive for the Evolvement and Development of Lung Adenocarcinoma,” Cancer Letters 582 (2024): 216588. 38097132 10.1016/j.canlet.2023.216588

[29] F. Zhang , J. Jiang , H. Qian , Y. Yan , and W. Xu , “Exosomal circRNA: Emerging Insights Into Cancer Progression and Clinical Application Potential,” Journal of Hematology & Oncology 16 (2023): 67. 37365670 10.1186/s13045-023-01452-2 PMC10294326

[30] J. Gao , Y. Q. Ao , L. X. Zhang , et al., “Exosomal circZNF451 Restrains Anti‐PD1 Treatment in Lung Adenocarcinoma via Polarizing Macrophages by Complexing With TRIM56 and FXR1,” Journal of Experimental & Clinical Cancer Rresearch: CR 41 (2022): 295. 10.1186/s13046-022-02505-z PMC9547453 36209117

[31] Y. Chen , X. Liu , Y. Li , C. Quan , L. Zheng , and K. Huang , “Lung Cancer Therapy Targeting Histone Methylation: Opportunities and Challenges,” Computational and Structural Biotechnology Journal 16 (2018): 211–223. 30002791 10.1016/j.csbj.2018.06.001 PMC6039709

[32] N. Zhang , G. Y. Song , Q. H. Yu , et al., “Evaluation of the lncRNA‐miRNA‐mRNA ceRNA Network in Lungs of miR‐147 (−/−) Mice,” Frontiers in Pharmacology 15 (2024): 1335374. 38510653 10.3389/fphar.2024.1335374 PMC10953689

[33] M. G. Gold , T. Gonen , and J. D. Scott , “Local cAMP Signaling in Disease at a Glance,” Journal of Cell Science 126 (2013): 4537–4543. 24124191 10.1242/jcs.133751 PMC3795333

[34] Y. Wang , R. Zuo , G. Huo , et al., “TWF1 Induces Autophagy and Accelerates Malignant Phenotype in Lung Adenocarcinoma via Inhibiting the cAMP Signaling Pathway,” FASEB Journal : Official Publication of the Federation of American Societies for Experimental Biology 37 (2023): e23051. 37358822 10.1096/fj.202300248R

[35] T. Zou , J. Liu , L. She , et al., “A Perspective Profile of ADCY1 in cAMP Signaling With Drug‐Resistance in Lung Cancer,” Journal of Cancer 10 (2019): 6848–6857. 31839819 10.7150/jca.36614 PMC6909948

[36] F. Murray and P. A. Insel , “Targeting cAMP in Chronic Lymphocytic Leukemia: A Pathway‐Dependent Approach for the Treatment of Leukemia and Lymphoma,” Expert Opinion on Therapeutic Targets 17 (2013): 937–949. 23647244 10.1517/14728222.2013.798304

[37] Q. Zheng , S. Min , and Q. Zhou , “Identification of Potential Diagnostic and Prognostic Biomarkers for LUAD Based on TCGA and GEO Databases,” Bioscience Reports 41 (2021): 1–24. 10.1042/BSR20204370 PMC8182989 34017995

[38] H. Rq , L. Xj , L. Liang , et al., “The Suppressive Role of miR‐542‐5p in NSCLC: The Evidence From Clinical Data and in Vivo Validation Using a Chick Chorioallantoic Membrane Model,” BMC Cancer 17 (2017): 1–15. 28927388 10.1186/s12885-017-3646-1 PMC5606087

[39] E. J. Kim and Y. S. Juhnn , “Cyclic AMP Signaling Reduces Sirtuin 6 Expression in Non‐Small Cell Lung Cancer Cells by Promoting Ubiquitin‐Proteasomal Degradation via Inhibition of the Raf‐MEK‐ERK (Raf/Mitogen‐Activated Extracellular Signal‐Regulated Kinase/Extracellular Signal‐Regulated Kinase) Pathway,” Journal of Biological Chemistry 290 (2015): 9604–9613. 25713071 10.1074/jbc.M114.633198 PMC4392263

[40] T. Sang , Y. Wang , Z. Wang , et al., “NEAT1 Deficiency Promotes Corneal Epithelial Wound Healing by Activating cAMP Signaling Pathway,” Investigative Ophthalmology & Visual Science 65 (2024): 10. 10.1167/iovs.65.3.10 PMC10929751 38466291

[41] X. H. Liang , W. B. Deng , Y. F. Liu , et al., “Non‐Coding RNA LINC00473 Mediates Decidualization of Human Endometrial Stromal Cells in Response to cAMP Signaling,” Scientific Reports 6 (2016): 22744. 26947914 10.1038/srep22744 PMC4780002

[42] P. Lu , J. Fan , B. Li , X. Wang , and M. Song , “A Novel Protein Encoded by circLARP1B Promotes the Proliferation and Migration of Vascular Smooth Muscle Cells by Suppressing cAMP Signaling,” Atherosclerosis 395 (2024): 117575. 38851155 10.1016/j.atherosclerosis.2024.117575

[43] X. Lv , L. Yang , Y. Xie , and M. R. Momeni , “Non‐Coding RNAs and Exosomal Non‐Coding RNAs in Lung Cancer: Insights Into Their Functions,” Frontiers in Cell and Development Biology 12 (2024): 1397788. 10.3389/fcell.2024.1397788 PMC11163066 38859962

[44] D. Chu , L. Chen , W. Li , and H. Zhang , “An Exosomes‐Related lncRNA Prognostic Model Correlates With the Immune Microenvironment and Therapy Response in Lung Adenocarcinoma,” Clinical and Experimental Medicine 24 (2024): 104. 38761234 10.1007/s10238-024-01319-x PMC11102376

[45] H. Zhao , H. Wei , J. He , et al., “Propofol Disrupts Cell Carcinogenesis and Aerobic Glycolysis by Regulating circTADA2A/miR‐455‐3p/FOXM1 Axis in Lung Cancer,” Cell Cycle (Georgetown, Texas) 19 (2020): 2538–2552. 10.1080/15384101.2020.1810393 PMC7553507 32857667

[46] J. Zhang , X. Ma , R. Zhou , and Y. Zhou , “TRPS1 and YAP1 Regulate Cell Proliferation and Drug Resistance of Osteosarcoma via Competitively Binding to the Target of circTADA2A ‐ miR‐129‐5p,” Oncotargets and Therapy 13 (2020): 12397–12407. 33293831 10.2147/OTT.S276953 PMC7719346

[47] J. Cui , W. Li , G. Liu , et al., “A Novel Circular RNA, hsa_circ_0043278, Acts as a Potential Biomarker and Promotes Non‐Small Cell Lung Cancer Cell Proliferation and Migration by Regulating miR‐520f,” Artificial Cells, Nanomedicine, and Biotechnology 47 (2019): 810–821. 30873868 10.1080/21691401.2019.1575847

[48] Z. Li , H. Yao , S. Wang , G. Li , and X. Gu , “CircTADA2A Suppresses the Progression of Colorectal Cancer via miR‐374a‐3p/KLF14 Axis,” Journal of Experimental & Clinical Cancer Rresearch: CR 39 (2020): 160. 10.1186/s13046-020-01642-7 PMC7429896 32799891

[49] L. Li , M. Peng , W. Xue , et al., “Integrated Analysis of Dysregulated Long Non‐Coding RNAs/microRNAs/mRNAs in Metastasis of Lung Adenocarcinoma,” Journal of Translational Medicine 16 (2018): 372. 30587197 10.1186/s12967-018-1732-z PMC6307237

[50] Z. Chen , X. Ma , Z. Chen , et al., “Exosome‐Transported circ_0061407 and circ_0008103 Play a Tumour‐Repressive Role and Show Diagnostic Value in Non‐Small‐Cell Lung Cancer,” Journal of Translational Medicine 22 (2024): 427. 38711144 10.1186/s12967-024-05215-6 PMC11071259

[51] Y. Kang , J. You , Y. Gan , et al., “Serum and Serum Exosomal CircRNAs hsa_circ_0001492, hsa_circ_0001439, and hsa_circ_0000896 as Diagnostic Biomarkers for Lung Adenocarcinoma,” Frontiers in Oncology 12 (2022): 912246. 35747792 10.3389/fonc.2022.912246 PMC9209657

